# Detection of Antiphosphatidylserine/Prothrombin Antibodies and Their Potential Diagnostic Value

**DOI:** 10.1155/2013/724592

**Published:** 2013-09-26

**Authors:** Polona Žigon, Saša Čučnik, Aleš Ambrožič, Tanja Kveder, Snežna Sodin Šemrl, Blaž Rozman, Borut Božič

**Affiliations:** ^1^Immunology Laboratory, Department of Rheumatology, University Medical Centre, 1000 Ljubljana, Slovenia; ^2^Faculty of Mathematics, Natural Science and Information Technology, University of Primorska, 6000 Koper, Slovenia; ^3^Faculty of Pharmacy, University of Ljubljana, 1000 Ljubljana, Slovenia

## Abstract

Antiprothrombin antibodies, measured with phosphatidylserine/prothrombin complex (aPS/PT) ELISA, have been reported to be associated with antiphospholipid syndrome (APS). They are currently being evaluated as a potential classification criterion for this autoimmune disease, characterized by thromboses and obstetric complications. Given the present lack of clinically useful tests for the accurate diagnosis of APS, we aimed to evaluate *in-house* and commercial assays for determination of aPS/PT as a potential serological marker for APS. We screened 156 patients with systemic autoimmune diseases for antibodies against PS/PT, **β**_2_-glycoprotein I, cardiolipin and for lupus anticoagulant activity. We demonstrated a high degree of concordance between the concentrations of aPS/PT measured with the *in-house* and commercial assays. Both assays performed comparably relating to the clinical manifestations of APS, such as arterial and venous thromboses and obstetric complications. IgG aPS/PT represented the strongest independent risk factor for the presence of obstetric complications, among all tested aPL. Both IgG and IgM aPS/PT were associated with venous thrombosis, but not with arterial thrombosis. Most importantly, the association between the presence of IgG/IgM aPS/PT and lupus anticoagulant activity was highly significant. Taken together, aPS/PT antibodies detected with the *in-house* or commercial ELISA represent a promising serological marker for APS and its subsets.

## 1. Introduction

Antiphospholipid syndrome (APS) is an autoimmune disease identified by clinical manifestations of vascular thromboses and obstetric complications, together with the serology of persistently positive antiphospholipid antibodies (aPL) [1, 2]. aPL represent a heterogeneous group of immunoglobulins detected by coagulation tests, such as lupus anticoagulant activity (LA) or measured by an enzyme-linked immunosorbent assays (ELISAs) as anticardiolipin antibodies (aCL) or antibodies against *β*_2_-glycoprotein I (anti-*β*_2_GPI). 

Antiprothrombin antibodies have not yet been included in the classification criteria of APS, although they are emerging as an increasingly important supportive marker. In recent years, their association with APS was evaluated with contradictory outcomes. Some studies failed to reveal a significant association of antiprothrombin antibodies with manifestations of APS [3–6], yet in other studies, their correlation to APS was found. The possibility of antiprothrombin antibodies becoming an additional serological classification criterion for APS emerged, especially relevant in APS patients negative for classical aPL [7–10]. 

Antibodies recognizing prothrombin can be detected by ELISA targeting prothrombin alone, coated onto irradiated plates (aPT), or targeting the phosphatidylserine/prothrombin complex (aPS/PT). It was demonstrated that antibodies recognized prothrombin more efficiently in aPS/PT ELISA [11] and that aPS/PT correlated better with APS and LA activity [7, 8, 12, 13] as compared to aPT. The inclusion of aPS/PT, but not aPT, to the laboratory criteria for APS has been proposed [14]. The first published aPS/PT protocol [7] was later modified in our previous study [10] in order to increase the analytical sensitivity of the test. We have reported that our *in-house* aPS/PT ELISA was the most optimal method for the determination of all clinically relevant aPS/PT antibodies, exhibiting the highest percentage of LA activity, compared to aCL and anti-*β*_2_GPI [10, 15]. We reported different avidity of antiprothrombin antibodies, as it is also known for several other autoimmune antibodies [16–18]. Moreover, we showed that the avidity was associated with their detection by different ELISAs. 

Until recently, only some aPT commercial kits were available and they showed poor diagnostic sensitivity and specificity [6]. In 2010, the *commercial QUANTA Lite* aPS/PT IgG/IgM and LAC assays became available as an aid in the diagnosis of APS. 

The lack of comparative analytical data between the various aPS/PT assays led the present investigation to compare our *in-house* aPS/PT ELISA with the commercial *QUANTA Lite* aPS/PT assay, in terms of diagnostic efficiency of aPS/PT. We aimed to determine whether the presence of aPS/PT antibodies was associated with specific clinical manifestation of APS and whether they could therefore become an additional serological marker of APS diagnosis. Additionally, our goal was to compare commercial kits enabling the detection of low avidity antiprothrombin antibodies, as was previously shown for our *in-house* aPS/PT ELISA [10].

## 2. Materials and Methods

### 2.1. Subjects

Sera from 156 of patients with systemic autoimmune diseases (34 males and 122 females, mean age 47 years, range 16–85) were analyzed in a cross-sectional study. APS, based on the revised International Consensus criteria [1], was diagnosed in 58 patients, APS associated with systemic lupus erythematosus (SLE) [19] in 38 patients. The control groups of patients were comprised of 24 patients with SLE, 25 patients with rheumatoid arthritis (RA) [20], and 11 Sjögren's syndrome patients (SS) [21]. Among all, 42 patients experienced an arterial event, 53 had a venous event, and 28 had obstetric complications (Table 1). The patients had their sera collected and analyzed when they were examined at the Department of Rheumatology (University Medical Centre, Ljubljana). This study was conducted as part of the National Research Program titled “Systemic Autoimmune Diseases” (number P3-0314). Participants signed an informed consent and the study was approved by the National Medical Ethics Committee, Ljubljana, Slovenia.

### 2.2. *In-House* aPS/PT ELISA

The levels of aPS/PT were detected according to the previously described aPS/PT ELISA protocol [10]. Medium binding plates (Costar, Cambridge, USA) were coated with phosphatidylserine in chloroform/methanol 1 : 4 and dried overnight at 4°C. Following blocking with Tris-buffered saline (TBS) containing 1% bovine serum albumin (BSA) and 5 mM CaCl_2_ (1% BSA/TBS-Ca), 25 *μ*L of human prothrombin (Enzyme Research Laboratories, Ltd., Swansea, UK) (20 mg/L) and 25 *μ*L of patients' sera diluted 1 : 50 were applied to wells immediately one after the other and incubated for 1 h at room temperature. After that, alkaline phosphatase-conjugated goat anti-human IgG or IgM (ACSC, Westbury, USA) were applied in TBS/Tween (0.05% Tween) and incubated for 30 min. Following 4 washes in TBS/Tween, 100 *μ*L/well of para-nitrophenylphosphate (Sigma Chemical Company, St. Louis, USA) in diethanolamine buffer (pH 9.8) was applied and OD_405_ was kinetically measured by a spectrometer (Tecan Sunrise Remote, Grödig, Austria). 

### 2.3. INOVA *QUANTA Lite* aPS/PT ELISA

A semiquantitative ELISA for the individual detection of IgG and IgM aPS/PT was performed following the manufacturer's instruction (INOVA Diagnostics, CA, USA).

### 2.4. INOVA *QUANTA Lite* LAC ELISA

A semiquantitative ELISA for the detection of both IgG and IgM aPS/PT class antibodies was performed following the manufacturer's instructions (INOVA Diagnostics, CA, USA).

### 2.5. aCL ELISA and Anti-*β*_2_GPI ELISA

IgG and IgM aCL were determined according to the previously described method [22, 23]. Anti-*β*_2_GPI were measured with our *in-house* ELISA [24] and evaluated through the European forum for aPL [25].

### 2.6. Avidity Determination of IgG aPS/PT by Chaotropic aPS/PT ELISA

The chaotropic aPS/PT ELISA with increased concentrations of NaCl during the antibody binding phase was used for avidity determination [10, 15]. The presence of high avidity aPS/PT antibodies was identified when the binding of antibodies at 0.5 M NaCl remained higher than 70% of the initial binding at 0.136 M NaCl. Low avidity aPS/PT antibodies were declared when the binding decreased ≤30% of the initial binding. The remaining samples were considered to be of heterogeneous avidity. 

### 2.7. Lupus Anticoagulant

The assay was performed in blood samples collected in tubes containing 0.109 M sodium citrate. Platelet-poor plasma was obtained by centrifugation at 2400 g for 20 min at 4°C. After filtration, aliquots were stored at −80°C until use. Clotting tests were performed using coagulation analyzer BCS Siemens according to the previous guidelines of the International Society on Thrombosis and Haemostasis ISTH [26]. Simplified Dilute Russell's Viper Venom Test (dRVVT) was performed using LA1 Screening reagent and LA2 Confirmatory reagent (Siemens) following the manufacturer's instructions [27]. A dRVVT ratio (LA1 screen/LA2 confirmation) above 1.2 was considered positive for LA activity. Activity of LA was quantified as follows: low positive (LA1/LA2 = 1.2–1.5), medium (LA1/LA2 = 1.5–2.0), and high positive (LA1/LA2 > 2.0).

### 2.8. Statistical Analysis

Statistical analysis was performed using the SPSS 15.0 program. Normal distribution was evaluated using descriptive statistic parameters, curve fittings, and Kolmogorov-Smirnov test. The Receiver Operating Characteristic (ROC) analysis and the area under the curve (AUC) were used to assess the diagnostic performance of the measured marker(s). The results of multivariate logistic models were approximated by odds ratio with its 95% confidence interval (OR (95%)). A 2-sided *P* value < 0.05 was considered statistically significant.

## 3. Results

### 3.1. Correlation between *In-House* and *QUANTA Lite* aPS/PT Assays

**Figure 1:**
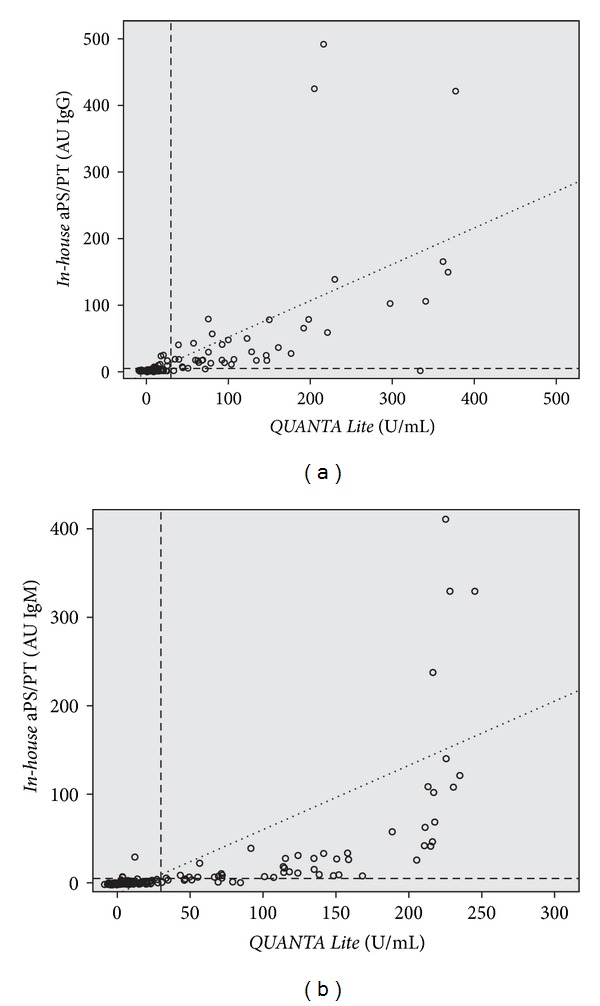
aPS/PT antibodies detected with the *in-house* ELISA correlated significantly with results of *QUANTA Lite* IgG (a) and IgM (b) in 156 patient sera. The dashed lines represent the cut-off value (*in-house* ELISA 5 AU, *QUANTA Lite* 30 U/mL). AU: arbitrary units.

Both IgG and IgM aPS/PT antibodies detected with *in-house* ELISA correlated significantly with results of *QUANTA Lite* immunoassays using Spearman correlation (rho = 0.744 for IgG; rho = 0.865 for IgM) (Figures 1(a) and 1(b)) in 156 patient sera. Substantial concordance was validated also with Lin's concordance correlation coefficient (Rc = 0.625 for IgG and Rc = 0.572 for IgM), which is a reproducibility measure.

### 3.2. Diagnostic Applicability Comparison of Different Antiphospholipid Antibody Assays

**Figure 2:**
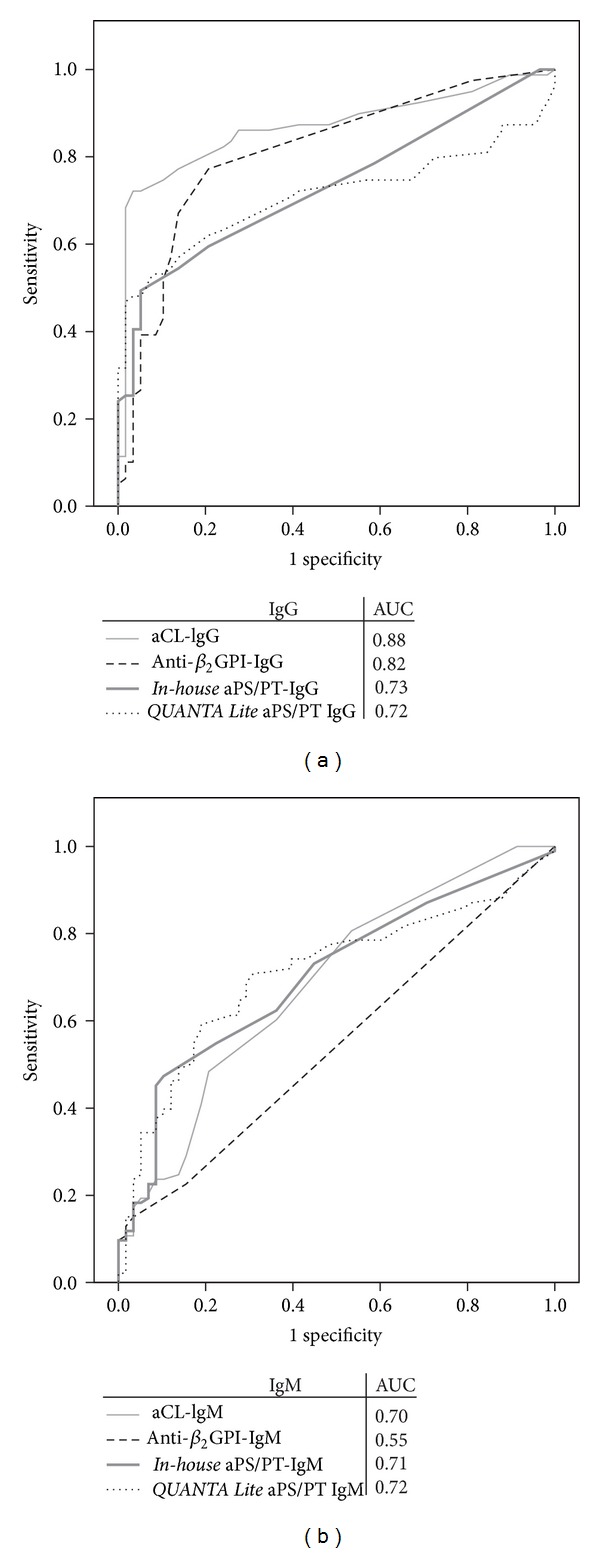
Receiver Operating Characteristic (ROC) curves and area under the curve (AUC) of different antiphospholipid antibody methods for APS (*n* = 156). The higher values of AUC indicate better diagnostic efficiency of the test. aCL: anticardiolipin, anti-*β*_2_GPI: anti-*β*_2_glycoprotein, and aPS/PT antiphosphatidylserine/prothrombin.

We evaluated APS diagnostic applicability of all assays with a receiver operating characteristic curve (ROC curve) and estimated the area under the curve (Figure 2). The highest diagnostic efficiency for APS was achieved by aCL IgG (AUC = 0.88) (Figure 2(a)). Both, the *in-house* and the *QUANTA Lite, IgG aPS/PT* methods were comparable (0.73 and 0.72, resp.). All methods detecting IgM aPL (Figure 2(b)) showed a lower overall performance compared to IgG aCL.

### 3.3. Relationship of aPL with Thrombosis and Obstetric Manifestations

The positivity of an individual aPL test and clinical manifestations of APS were considered in a logistic regression analysis (Table 2). IgG and IgM aPS/PT measured with the *in-house* and *QUANTA Lite* ELISA presented the highest independent risk factor for obstetric complication, among all tested aPL (OR = 9.3 and OR = 6.3, resp., for IgG and IgM). Both IgG and IgM aPS/PT measured with either assay were an independent risk factor for the presence of venous thrombosis. However, the highest risk for venous thrombosis was achieved by LA (OR = 5.6). IgG aPS/PT measured with *QUANTA Lite* ELISA were also an independent risk factor for the presence of arterial thrombosis, but the association was rather weak (OR = 2.3, *P* = 0.03).

The *QUANTA Lite* LAC screen test detected all sera positive in the individual IgG or IgM aPS/PT assays. The LAC screen did not achieve the diagnostic efficiency of the established LA coagulation test (Table 2).

### 3.4. Relationship between LA and aCL, Anti-*β*_2_GPI, or aPS/PT Antibodies

Out of 156 patients included in the study, 16 (10%) did not have their LA activity determined due to their anticoagulant treatment. Seven patients (3 APS, 3 RA, and 1 SS) were solely positive for LA, three of them had low, and four medium LA activity. Among all the aCL positive patients, 51% had LA; among the anti-*β*_2_GPI positive patients, 55% had LA, while among the aPS/PT positive patients, 66% had LA activity. aPS/PT, measured with either *in-house* or commercial assay, were much higher independent risk factors for the presence of LA activity (OR > 15.3 for IgG and OR > 12.9 for IgM) as compared to either aCL or anti-*β*_2_GPI (OR < 9.0 for IgG and OR < 4.6 for IgM, resp.) (Table 3).

### 3.5. Avidity of aPS/PT

Avidity of IgG aPS/PT was determined using a chaotropic IgG aPS/PT ELISA in aPS/PT positive patients detected by the *in-house* IgG aPS/PT ELISA, regardless of the antibody level. Antibodies were detected of predominantly low, heterogeneous, and predominantly high avidity (*n* = 9, 33, 9 out of 51, resp.). Both the *QUANTA Lite* IgG aPS/PT ELISA and LAC screen assays detected more than 40% of sera with low avidity antibodies and more than 85% of those with heterogeneous or high avidity aPS/PT (Table 4).

Among nine patients with low avidity aPS/PT, seven were diagnosed with APS; three of which experienced arterial thrombosis, five venous thrombosis, and one had obstetric complications. Two out of 97 APS patients included in the study were positive in the *QUANTA Lite* IgG aPS/PT ELISA, but negative in the *in-house* aPS/PT ELISA and their avidity was not determined.

## 4. Discussion

A comprehensive comparative study of anti-prothrombin antibodies (on two *in-house* and three commercial aPT tests) conducted in 2007 by Tincani et al. reported issues with reproducibility and interpretation of results and advised against their routine use [6]. Antibodies against PS/PT were first described by Matsuda et al. in patients with LA in 1996 [28], while one year later, Galli et al. [11] reported that the aPS/PT assay was more sensitive than the aPT test. In 2000, Atsumi et al. pointed out that aPS/PT can be used not only to confirm the presence of LA, but also to serve (in addition to aCL and anti-*β*_2_GPI) as one of the markers of APS and also thrombotic events in patients with autoimmune diseases [7]. Since 2000, aPS/PT antibody detection stood the test of time and was proven as a useful tool for the diagnosis of APS [29]. Comparative studies gave additional indication of their diagnostic relevance and confirmed their closer correlation with APS and LA activity as compared to aPT [7, 8, 13, 30]. The protocol by Matsuda et al. [12] used a higher concentration of phosphatidylserine (65 *μ*g/mL) and prothrombin (20 *μ*g/mL), while the protocols by Atsumi et al. [7], Tincani et al. [6], and Žigon et al. [10] all used lower concentrations of phosphatidylserine (50 *μ*g/mL) and prothrombin (10 *μ*g/mL). Additional major modifications between Matsuda et al. and later protocols are different times and temperatures of phosphatidylserine and prothrombin incubation. Recently, we have reported that our modified *in-house* aPS/PT ELISA (with increased analytical sensitivity) detects both presumably different populations of antibodies and low avidity antibodies, as well as it enables the identification of patients negative for other anti-phospholipid antibodies [10]. The modification (by means of the concomitant antigen and antibody incubation) resulted in increased prothrombin concentration on phospholipid surface and possible exposure of additional epitopes on prothrombin, enabling a higher intensity of antiprothrombin antibody binding. 

The current report shows that aPS/PT antibodies were the strongest independent risk factor for obstetric complications in our population of patients. Two previous studies on females with obstetric disorders found that aPS/PT antibodies were not frequent in patients with unexplained recurrent miscarriages without APS [5, 31]. However, in a recent study, comprising 163 women negative for the classical repertoire of aPL, antiprothrombin antibodies appeared to be associated with previous adverse pregnancy outcome [32]; however, the author did not support the potential use of these antibodies in clinical practice. In our study, among 28 female patients (26 were diagnosed with APS), IgG aPS/PT antibodies (measured by either *in-house* or commercial assay) showed the strongest correlation with obstetric complications, among all aPL antibodies. Further studies are warranted on a larger population of obstetric patients. Taking into account the logistic regression results, IgG/IgM aPS/PT were also independent risk factors for the presence of venous thrombosis (OR = 3.5 and OR = 2.2, resp.). The highest OR for venous thrombosis is presented by LA (OR = 5.6), while all of the measured aPL showed statistically significantly correlation. On the other hand, aPS/PT antibodies were not strong independent risk factor for arterial thrombosis. Similarly, Vlagea et al. found no association between the presence of aPS/PT and arterial thrombosis [33]. Two previous studies [7, 8] have shown anti-prothrombin antibodies as an independent risk factor for arterial thrombosis, but other reports [6, 9, 34] presented their data without differentiating between arterial or venous thromboembolic events. In general, all IgM antibody subtypes of aPL demonstrated a lower diagnostic efficiency for thrombosis as compared to IgG aPL. These data are in line with the results of a systematic review [35] and a later study [36] which reported IgM aCL, anti-*β*_2_GPI, and anti-prothrombin antibodies to be less often associated with clinical events of APS than IgG. 

Correlation between aPS/PT and LA activity has been reported previously [7, 13, 33, 37] and our current study confirmed a strong correlation between aPS/PT and LA activity. IgG/IgM aPS/PT were the highest independent risk factors for LA activity with an OR > 12.9 as compared to aCL and anti-*β*_2_GPI with an OR < 9.0. Despite internationally accepted guidelines and many efforts to improve the standardization of LA activity assays, accurate detection and intralaboratory reproducibility are still not fully achieved. LA determination is a sequential series of analyses, which requires careful treatment of plasma specimens obtained from patients who are not receiving any anticoagulant therapy. So, the aPS/PT assay could represent a solid additional test performed using sera samples of patients regardless of anticoagulant therapy. 

Very few studies have reported on the avidity of anti-prothrombin antibodies. Avidity was shown to importantly influence positivity in different antiprothrombin ELISAs given that none of the low avidity antibodies were positive in the aPT ELISA [15]. On the contrary, aPS/PT assay enables the detection of low avidity antibodies, as evidenced in the current report. Our *in-house* IgG aPS/PT detected 9 positive patients with low avidity; however, *QUANTA Lite* IgG aPS/PT assay detected only 40% of low avidity anti-prothrombin antibodies. (Table 4). Our group has previously shown that avidity of anti-*β*_2_GPI importantly correlated with the clinical onset; therefore, it appeared reasonable to assume the same for the avidity of antiprothrombin antibodies [16]. However, we could not draw the same conclusion, but found that out of nine patients with low avidity aPS/PT antibodies, seven had APS. Therefore, a method enabling the detection of low avidity aPS/PT is also essential for possible inclusion of aPS/PT in the classification criteria for APS.

In conclusion, the present study is in line with the recommendations advising confirmation of previous data for “noncriteria aPL,” such as aPS/PT [38]. The only commercially available aPS/PT assay was evaluated in comparison to our *in-house* aPS/PT. aPS/PT detected with either *in-house* aPS/PT ELISA or with *QUANTA Lite* aPS/PT ELISA showed very high specificity for APS that could serve as an additional serological diagnostic marker for venous thrombosis and obstetric complications. The association of aPS/PT with LA activity was the highest among all aPL tested and therefore can be a useful feature of these antibodies. In summary, aPS/PT, measured with either *in-house* or commercial assay, in addition to aCL and anti-*β*_2_GPI antibodies, could represent an additional marker in patients with clinical manifestations of APS.

## Figures and Tables

**Table 1:** Prevalence of arterial thrombosis, venous thrombosis, and obstetric complications in the groups of selected autoimmune patients.

	No. (f/m)	Arterial thrombosis (41)	Venous thrombosis (53)	Obstetric complications (28)	Total
APS	**58 **(34/24)	**21** (13/8)	**33 **(16/17)	**12**	**97**
APS + SLE	**38** (31/7)	**16 **(14/2)	**17 **(11/6)	**14**	

SLE	**24 **(24/0)	**4** (4/0)	**1 **(1/0)	**1**	**60**
RA	**25 **(22/3)	**0**	**2 **(2/0)	**0**	
SS	**11** (11/0)	**0**	**0**	**1**	

No.: number of patients, f/m: female/male, APS: antiphospholipid syndrome, RA: rheumatoid arthritis, SLE: systemic lupus erythematosus, and SS: Sjögren's syndrome.

**Table 2:** Antiphospholipid antibodies and LA in a relationship to arterial thrombosis (AT), venous thrombosis (VT), and obstetric complications (OC).

Antibody	Arterial thrombosis (41)					
		No.	*P* value	Odds ratio (95% Cl)	Sensitivity %	Specificity %
LA		17	0.43	1.4 (0.6–2.9)	49	59
aCL	IgG	34	<0.001	**5.5 (2.3–13.4)**	83	53
	IgM	6	0.67	1.2 (0.4–3.5)	15	88
Anti-*β*_2_GPI	IgG	26	0.02	2.4 (1.2–5.1)	63	58
	IgM	5	0.99	1.0 (0.3–2.9)	12	88
*In-house* aPS/PT	IgG	18	0.08	1.9 (0.9–4.1)	44	71
	IgM	16	0.36	1.4 (0.6–2.9)	39	69
*QUANTA Lite* aPS/PT	IgG	17	0.03	2.3 (1.0–4.9)	41	77
	IgM	17	0.38	1.4 (0.7–2.9)	41	66
	LAC	26	0.50	1.3 (0.6–2.7)	62	43

	Venous thrombosis (53)					
LA		31	<0.001	**5.6 (2.6–12.2)**	70	70
aCL	IgG	39	<0.005	3.0 (1.5–6.2)	74	52
	IgM	12	0.010	3.5 (1.3–9.2)	23	92
Anti-*β*_2_GPI	IgG	35	<0.001	3.2 (1.6–6.5)	66	63
	IgM	10	0.070	2.5 (0.9–6.5)	19	91
*In-house* aPS/PT	IgG	27	<0.001	3.5 (1.7–7.0)	51	77
	IgM	24	0.021	2.2 (1.1–4.5)	45	73
*QUANTA Lite* aPS/PT	IgG	25	<0.001	4.0 (1.9–8.3)	47	82
	IgM	26	0.013	2.4 (1.2–4.7)	49	71
	LAC	37	0.043	2.1 (1.0–4.2)	70	47

	Obstetric complications (28)					
LA		13	<0.005	4.3 (1.6–11.9)	62	73
aCL	IgG	22	<0.001	5.8 (2.1–15.9)	79	61
	IgM	6	0.130	2.5 (0.8–7.8)	21	90
Anti-*β*_2_GPI	IgG	19	0.002	4.1 (1.7–10.4)	68	66
	IgM	5	0.278	2.0 (0.6–6.6)	18	90
*In-house* aPS/PT	IgG	18	<0.001	**9.3 (3.5–24.6)**	64	84
	IgM	15	<0.005	4.0 (1.6–9.9)	54	78
*QUANTA Lite* aPS/PT	IgG	14	<0.001	6.3 (2.4–16.7)	50	86
	IgM	16	<0.005	4.3 (1.7–10.6)	57	76
	LAC	21	0.042	2.7 (1.0–9.1)	75	48

aCL: anticardiolipin, anti-*β*_2_GPI: anti-*β*_2_glycoprotein, aPS/PT: anti-phosphatidylserine/prothrombin, LA: lupus anticoagulant, OR: odds ratio, and CI: confidence interval.

**Table 3:** Association between the presence of antiphospholipid antibodies and LA activity.

Antibody		Lupus anticoagulant activity	
		*P* value	Odds ratio (95% Cl)
aCL	IgG	<0.001	5.0 (2.4–10.6)
	IgM	<0.001	4.6 (1.6–13.7)
Anti-*β*_2_GPI	IgG	<0.001	9.0 (4.2–19.4)
	IgM	<0.001	1.2 (1.0–1.4)
*In-house* aPS/PT	IgG	<0.001	**21.6 (8.1–57.7)**
	IgM	<0.001	12.9 (5.4–30.6)
*QUANTA Lite* aPS/PT	IgG	<0.001	15.3 (5.8–40.5)
	IgM	<0.001	13.2 (5.6–30.8)
	LAC	<0.001	10.2 (4.4–23.6)

aCL: anticardiolipin, anti-*β*_2_GPI: anti-*β*_2_glycoprotein, and aPS/PT: antiphosphatidylserine/prothrombin.

**Table 4:** Association of aPS/PT avidity with clinical features of autoimmune patients.

aPS/PT avidity	Low (*n* = 9)	Heterogeneous (*n* = 33)	High (*n* = 9)
APS	7 (77%)	32 (97%)	9 (100%)
SLE	2	1	0
RA	0	0	0
SS	0	0	0
Thrombosis	8	26	8
Arterial	3	11	4
Venous	5	10	5
Obstetric disorder	1	14	4

aPS/PT positivity			
*In-house* IgG aPS/PT	9	33	9
*QUANTA Lite* IgG aPS/PT	4	29	8
*QUANTA Lite* LAC	7	32	9

APS: antiphospholipid syndrome, SLE: systemic lupus erythematosus, RA: rheumatoid arthritis, SS: Sjögren's syndrome, and aPS/PT: anti-phosphatidylserine/prothrombin antibodies.

## Abbreviations

Anti-*β*_2_GPI: Antibodies against *β*_2_-glycoprotein I
aCL: Anti-cardiolipin antibodies
APS: Antiphospholipid syndrome
aPS/PT: Phosphatidylserine-dependent anti-prothrombin antibodies
aPT: Antibodies against prothrombin alone
AUC: Area under the curve
CI: Confidence interval,
LA: Lupus anticoagulant
OR: Odds ratio
PS: Phosphatidylserine
RA: Rheumatoid arthritis
ROC: Receiver operating characteristic
SLE: Systemic lupus erythematosus
SS: Sjögren's syndrome.
